# Rationale and protocol for a cluster randomized pragmatic clinical trial testing behavioral economic implementation strategies to improve tobacco treatment rates for cancer patients who smoke

**DOI:** 10.1186/s13012-021-01139-7

**Published:** 2021-07-15

**Authors:** Brian P. Jenssen, Robert Schnoll, Rinad Beidas, Justin Bekelman, Anna-Marika Bauer, Callie Scott, Sarah Evers-Casey, Jody Nicoloso, Peter Gabriel, David A. Asch, Alison Buttenheim, Jessica Chen, Julissa Melo, Lawrence N. Shulman, Alicia B. W. Clifton, Adina Lieberman, Tasnim Salam, Kelly Zentgraf, Katharine A. Rendle, Krisda Chaiyachati, Rachel Shelton, E. Paul Wileyto, Sue Ware, Frank Leone

**Affiliations:** 1 Department of Pediatrics, Perelman School of Medicine, University of Pennsylvania, Philadelphia, USA; 2 Penn Center for Cancer Care Innovation, Abramson Cancer Center, Perelman School of Medicine, University of Pennsylvania, Philadelphia, USA; 3 Department of Psychiatry, Perelman School of Medicine, University of Pennsylvania, Philadelphia, USA; 4 Abramson Cancer Center, Perelman School of Medicine, University of Pennsylvania, Philadelphia, USA; 5 Center for Health Incentives and Behavioral Economics, University of Pennsylvania, Philadelphia, USA; 6 Penn Implementation Science Center (PISCE@LDI), Leonard Davis Institute of Health Economics, University of Pennsylvania, Philadelphia, USA; 7 Department of Radiation Oncology, Hospital of the University of Pennsylvania, Philadelphia, USA; 8 Department of Medical Ethics and Health Policy, Perelman School of Medicine, University of Pennsylvania, Philadelphia, USA; 9 Comprehensive Smoking Treatment Program, Perelman School of Medicine, University of Pennsylvania, Philadelphia, USA; 10 Division of General Internal Medicine, Department of Medicine, Perelman School of Medicine, University of Pennsylvania, Philadelphia, USA; 11 Department of Family Medicine and Community Health, Perelman School of Medicine, University of Pennsylvania, Philadelphia, USA; 12 University of Pennsylvania Health System, Philadelphia, USA; 13 Department of Sociomedical Sciences, Columbia University Mailman School of Public Health, New York, USA; 14 Center for Clinical Epidemiology and Biostatistics, Perelman School of Medicine, University of Pennsylvania, Philadelphia, USA; 15 Pulmonary, Allergy, & Critical Care Division, Perelman School of Medicine, University of Pennsylvania, Philadelphia, USA

**Keywords:** Tobacco use, Tobacco use treatment, Behavioral economics, Electronic health record, Pragmatic trials

## Abstract

**Background:**

Routine evidence-based tobacco use treatment minimizes cancer-specific and all-cause mortality, reduces treatment-related toxicity, and improves quality of life among patients receiving cancer care. Few cancer centers employ mechanisms to systematically refer patients to evidence-based tobacco cessation services. Implementation strategies informed by behavioral economics can increase tobacco use treatment engagement within oncology care.

**Methods:**

A four-arm cluster-randomized pragmatic trial will be conducted across nine clinical sites within the Implementation Science Center in Cancer Control Implementation Lab to compare the effect of behavioral economic implementation strategies delivered through embedded messages (or “nudges”) promoting patient engagement with the Tobacco Use Treatment Service (TUTS). Nudges are electronic medical record (EMR)-based messages delivered to patients, clinicians, or both, designed to counteract known patient and clinician biases that reduce treatment engagement. We used rapid cycle approaches (RCA) informed by relevant stakeholder experiences to refine and optimize our implementation strategies and methods prior to trial initiation. Data will be obtained via the EMR, clinician survey, and semi-structured interviews with a subset of clinicians and patients. The primary measure of implementation is penetration, defined as the TUTS referral rate. Secondary outcome measures of implementation include patient treatment engagement (defined as the number of patients who receive FDA-approved medication or behavioral counseling), quit attempts, and abstinence rates. The semi-structured interviews, guided by the Consolidated Framework for Implementation Research, will assess contextual factors and patient and clinician experiences with the nudges.

**Discussion:**

This study will be the first in the oncology setting to compare the effectiveness of nudges to clinicians and patients, both head-to-head and in combination, as implementation strategies to improve TUTS referral and engagement. We expect the study to (1) yield insights into the effectiveness of nudges as an implementation strategy to improve uptake of evidence-based tobacco use treatment within cancer care, and (2) advance our understanding of the multilevel contextual factors that drive response to these strategies. These results will lay the foundation for how patients with cancer who smoke are best engaged in tobacco use treatment and may lead to future research focused on scaling this approach across diverse centers.

**Trial registration:**

Clinicaltrials.gov, NCT04737031. Registered 3 February 2021.

Contributions to the literature

- This novel study, one of the Signature Pilot Projects (SPPs) within the Penn Implementation Science Center in Cancer Control (ISC3), is designed to evaluate behavioral economics-informed implementation strategies in the form of electronic medical record-based communications delivered to patients with cancer, clinicians specializing in cancer care, or both patients and clinicians to increase engagement in evidence-based tobacco use treatment.
- This study demonstrates the use of rapid cycle approaches and novel mixed methods to facilitate clinical trial implementation and enhance understanding of factors that influence the impact of implementation strategies on tobacco use treatment engagement.
- The results of this trial may lead to the dissemination of a low-cost and simple approach to increasing engagement of patients with cancer in tobacco use treatment, thereby maximizing the impact of oncologic care on patient health outcomes.

## Background

Continued tobacco smoking reduces survival among patients with cancer [1–3]. It accelerates tumor growth and increases disease progression, tumor resistance to treatment, and treatment-related toxicities [4–7]. Routinely delivered evidence-based tobacco use treatment (TUT) minimizes cancer-specific and all-cause mortality, reduces treatment-related toxicity, and improves quality of life [1]. Unfortunately, about 50% of cancer patients who smoked prior to their diagnosis continue to smoke after diagnosis and during treatment [8]. In response, the National Comprehensive Cancer Network [3], the American Society of Clinical Oncology [9], and the American Association for Cancer Research [10] have called for the implementation of evidence-based tobacco use treatment within oncology care. In 2015, TUT consisting of FDA-approved cessation medications and appropriate behavioral interventions received an “A” recommendation from the United States Preventive Services Task Force, given the high level of certainty of resulting benefit [11].

Despite the importance of TUT, only half of cancer centers consistently identify patient tobacco use [12] and few cancer centers employ systematic strategies to refer patients to evidence-based tobacco cessation services [11]. Acknowledging this gap, the National Cancer Institute (NCI) launched the Cancer Center Cessation Initiative (C3i) as part of the Cancer Moonshot to help centers develop effective ways to identify and engage patients who smoke [13]. One of the major objectives of C3i is to evaluate and overcome clinician, patient, clinic, and health system barriers by fully integrating TUT into cancer care services. C3i focuses on clinical workflow management and minimizing treatment plan variability as they relate to TUT.

As one of the first cancer centers selected in the initial C3i cohort, early efforts focused on the implementation of universal tobacco use screening and referral, based on the evidence-based *Ask-Advise-Connect* model [14]. Because lack of clinician experience with tobacco use treatment is a frequently cited barrier to implementation [15–17], our initial strategy used an automated opt-out “default” electronic medical record (EMR) referral to our Tobacco Use Treatment Service (TUTS). The initial intervention resulted in significant improvement in clinician engagement, with referral rates to TUTS rising from baseline 0 to 34% during the 6-month post-implementation period. While the experience suggested that clinician behavior was modifiable, over 60% of default referral orders were declined, implicating additional important barriers to change [18].

Implementation efforts to promote TUT engagement within oncology can be enhanced using strategies informed by behavioral economics, the application of which has shown potential to improve patient outcomes and transform healthcare delivery across a wide range of activities [19–22]. Prior work has identified specific barriers that prevent TUTS referral and engagement, including clinician pessimism regarding the ability to help patients stop using tobacco, misconceptions about patient resistance to treatment, and implicit biases regarding the capacity for patients to volitionally alter the course of illness [23]. These motivators are related to clinicians’ willingness to invest effort in help giving [24–26] and may prevent acquisition of new knowledge and skills [27]. From the patient perspective, several studies identify unique challenges faced by individuals with cancer when engaging in tobacco cessation efforts, including low self-efficacy, low perceived benefits of quitting, and perceived risk of treatment [28–30]. This trial was designed to evaluate the additional effectiveness of patient and clinician “nudges”—messages informed by behavioral economics designed to counteract known heuristics that reduce the likelihood of engaging in TUT—in a pragmatic and innovative way in order to increase TUTS referral.

Due to a variety of structural factors, both racial/ethnic minority and low socioeconomic status (SES) groups suffer disproportionately from the effects of tobacco marketing, have diminished access to evidence-based treatments for tobacco dependence, and report poorer response to smoking cessation treatments [31–35]. Thus, social inequities related to SES and race/ethnicity may play a role in determining referral and engagement decision-making, ultimately influencing health inequities. Examining these factors as potential effect modifiers is a particular focus in this trial. Further, a rapid-cycle approach to optimizing nudge framing prior to initiating the trial ensured efficient and effective use of informatics for participant recruitment, randomization, and nudge delivery. Directed nudges, fully integrated into the EMR, may represent a low-cost, simple, scalable, and effective implementation strategy for increasing TUT engagement among cancer patients, generalizable to other cancer treatment centers and settings, and improving cancer care outcomes.

## Methods

### Study aims

**Fig. 1:**
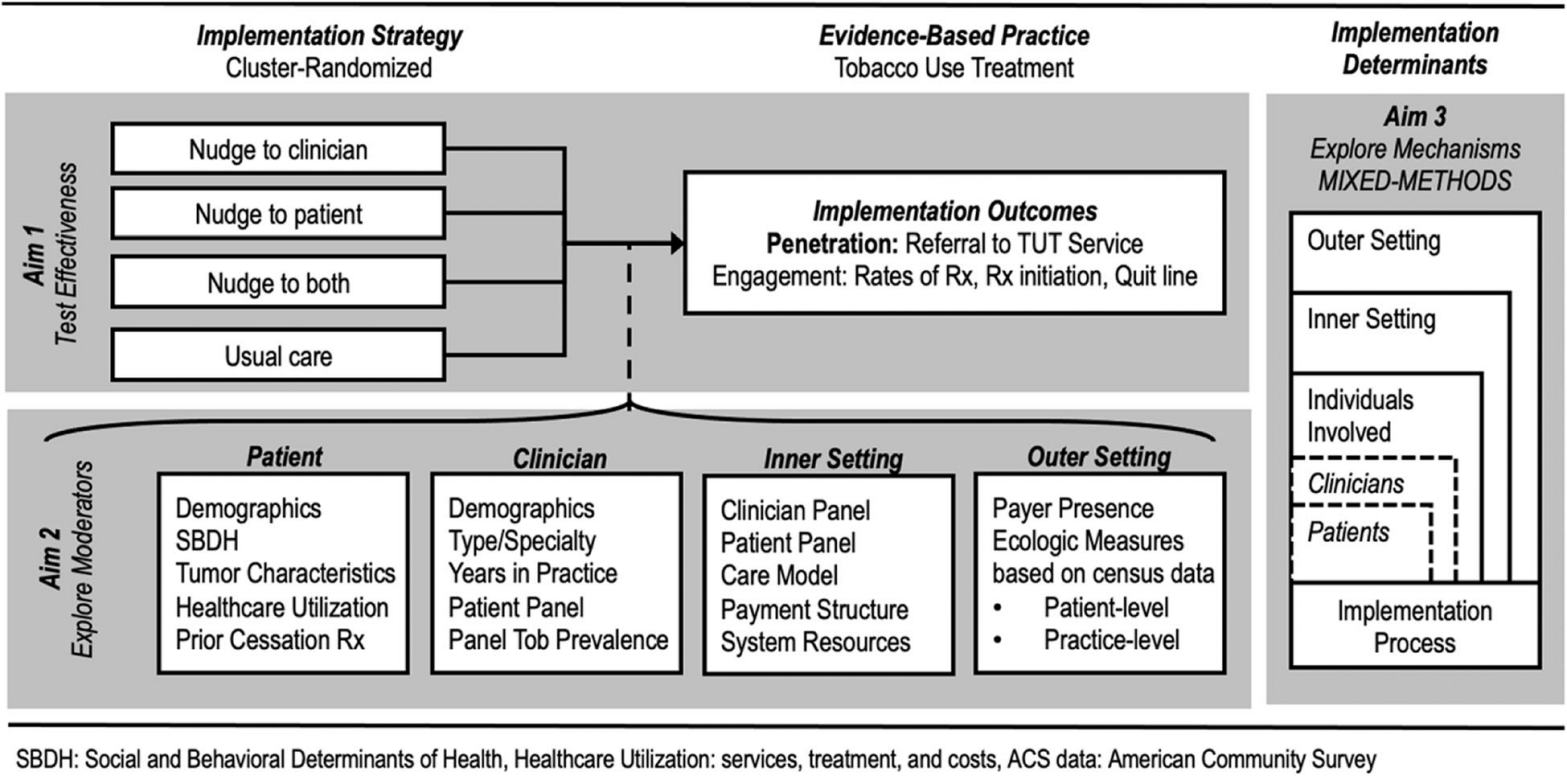
Study schema outlining the study aims

The trial incorporates three aims (see Fig. 1). First, the study will compare the effects of nudges directed at patients, clinicians, or both patients and clinicians, to usual care. Second, this study will examine patient and clinician characteristics, including patient characteristics that serve as indicators of equity, that moderate the impact of nudges on referral and engagement with evidence-based TUT. Lastly, this study seeks to understand the potential mechanisms by which our nudges increase TUTS referral and engagement.

### Study design

This study uses a four-arm cluster randomized pragmatic clinical trial design. Our sample of clinicians (N = 222) across our health system, referred to as our Implementation Lab (iLab; see below), will be randomized to one of the four treatment arms: clinician nudge, patient nudge, clinician and patient nudge, or usual care. Randomization is conducted by clusters, identified by paired connections between clinical coworkers within established networks (i.e., clusters) of interconnected colleagues (N = 95). Clusters were formed between clinicians with overlapping patient pools to reduce contamination between clusters. The clusters are not site-specific as many clinicians work at multiple sites. For patients (N = 900), assignment to the treatment arms is based upon the first clinician they are scheduled to see (i.e., the clinician for the index visit). The nudge is then delivered in conjunction with the next visit with any clinician in the same study arm. The primary outcome is the provision of a referral to the patient for treatment by the Tobacco Use Treatment Service (TUTS). Secondary outcomes include treatment engagement defined as the number of patients who receive FDA-approved medication or behavioral counseling for tobacco use, patient quit attempts, and patient abstinence rate. We hypothesize that each nudge will significantly increase TUTS referral and engagement compared to usual care, and that the combination of nudges to clinicians and to patients will be the most effective.

Our secondary objective is to conduct a quantitative evaluation using secondary data and a baseline survey (obtained via the EMR for patients and by a survey for clinicians) to identify effect modifiers of implementation effects on TUTS referral and engagement. We will explore variability in the nudge impact by clinician (e.g., years in practice, practice size), patient (e.g., race, sex), and inner setting factors (e.g., community vs. hospital setting).

For aim 3, we will conduct semi-structured interviews with a subset of participants (clinicians and patients), selected using purposive sampling to ensure that diverse perspectives are represented across important equity dimensions such as race/ethnicity, setting (i.e., urban vs non-urban), and neighborhood-level socioeconomic status, in order to increase understanding of potential mechanisms underlying the effects of nudges.

### Study setting, population, and duration

**Fig. 2:**
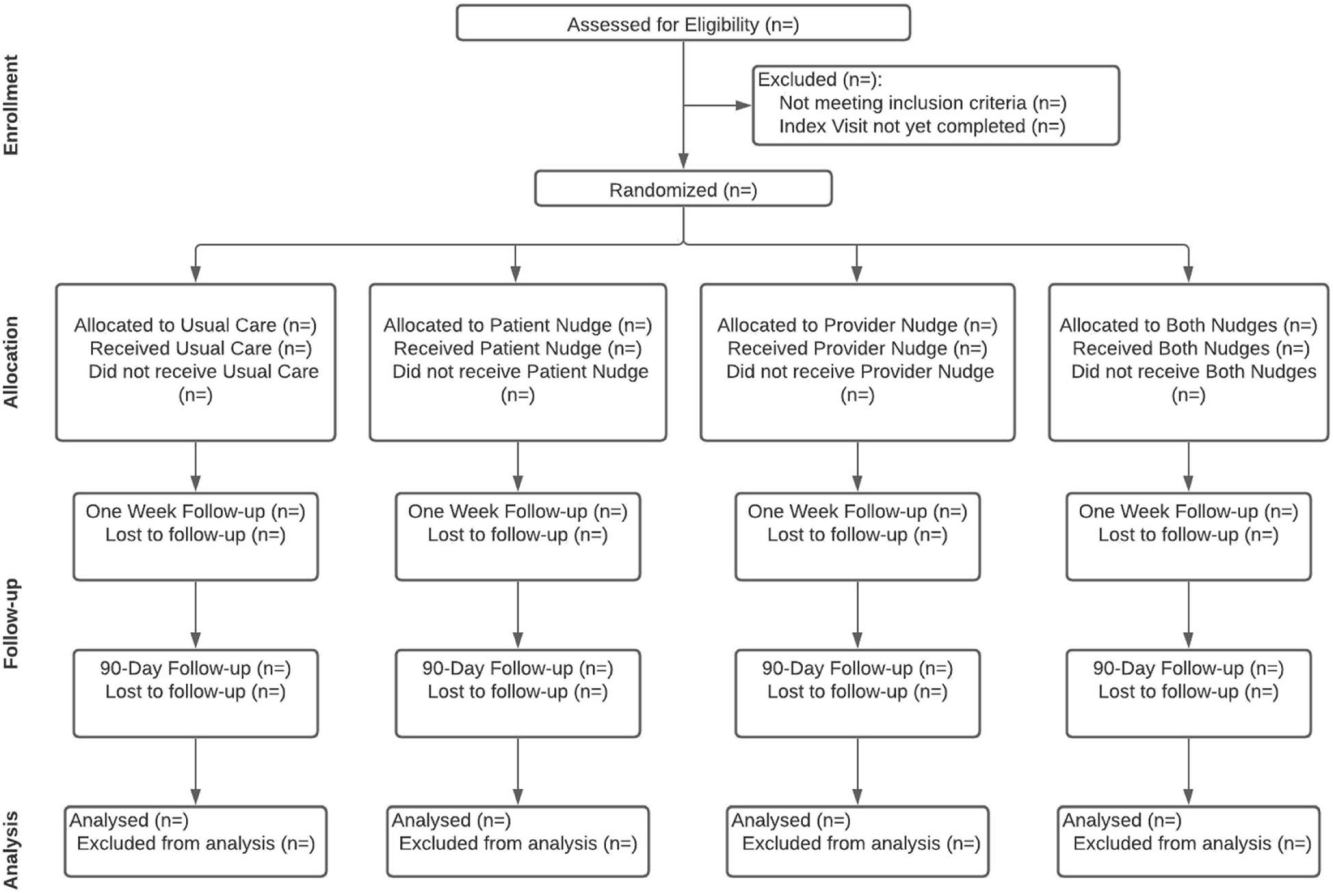
Consort diagram

We will conduct this study within our Implementation Science Center in Cancer Control (ISC3) Implementation Lab (iLab), which consists of cancer units at 5 hospitals and 4 regional clinics within Penn Medicine’s Abramson Cancer Center, which delivers cancer care to more than 20,000 patients each year. The clinician sample will include oncologists and Advanced Practice Providers, working within medical oncology, radiation oncology, and gynecologic oncology sites at the Hospital of the University of Pennsylvania, Pennsylvania Hospital, Penn Presbyterian Medical Center, Chester County Hospital, Lancaster General Hospital, Valley Forge Medical Center, Radnor Medical Center, Cherry Hill Medical Center, and Regional Hematology Oncology Associates. Eligibility criteria for clinician participants include (1) currently in practice at an iLab site; (2) prescribing authority in Pennsylvania or New Jersey (i.e., physician, nurse practitioner, physician assistant); (3) cared for at least 1 tobacco-using patient in 30 days prior to recruitment; and (4) English-speaking (messages will be in English). Eligibility criteria for patient participants include any patient diagnosed with cancer who reports current tobacco smoking (as assessed by staff collecting vital signs or initially rooming the patients such as nurses, front desk staff, medical assistants, or technicians during an index visit) and scheduled to visit with a clinician included in the study sample. Since this is a pragmatic trial, patients will accrue as they are seen by a sample clinician at a participating practice site. We anticipate that the trial will require about 6 months to accrue target samples. Once the patient visit occurs where the nudges are delivered (or not for those randomized to usual care), there will be a 90-day follow-up to assess engagement in TUT (see Fig. 2).

This study was approved by the University of Pennsylvania IRB, which covered approval at other sites under a reliance agreement. Since this is a pragmatic trial focused on improving implementation of evidence-based practices, the study represents a minimal risk to patients. Main study procedures in pursuit of aims 1 and 2 received a waiver of participant informed consent for both clinicians and patients. For aim 3, however, potential participants will be asked to provide informed consent prior to data collection.

### Overview of study procedures

**Fig. 3:**
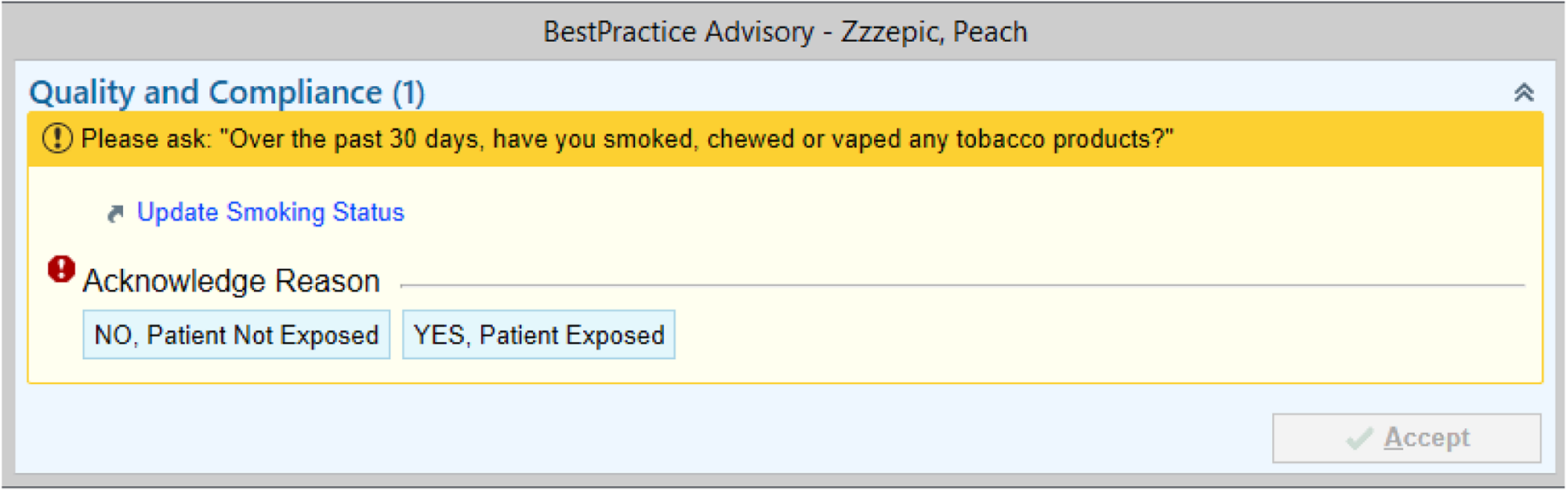
Best practice alert received by MAs during the index visit

All clinicians within our iLab were automatically enrolled into the study after an opportunity to opt out of participation. Patient enrollment begins with a positive tobacco use assessment conducted at the first patient visit within the study period, termed the *index visit*. Initial assessment of patient tobacco use utilizes a standardized Best Practice Alert (BPA) integrated into the EMR and found to be effective in our previous study [18]. This BPA is activated within the check-in and vital sign workflow, requiring assessment of tobacco exposure within the past 30 days, and is satisfied with one of two possible answers (see Fig. 3). Assignment to the study arm is based on the clinician for the index visit. The “subsequent visit” is then the next scheduled visit with a provider randomized to that same arm.

**Fig. 4:**
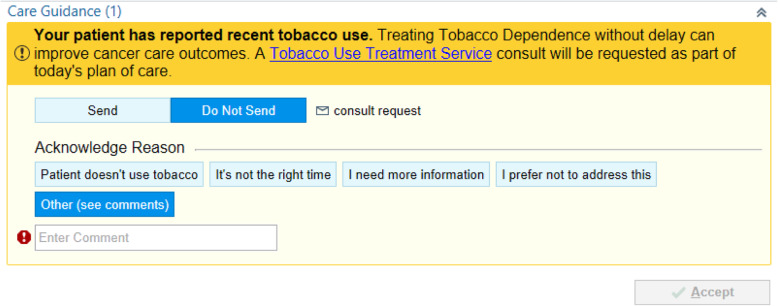
Best practice alert received by clinicians during the subsequent visit

**Fig. 5:**
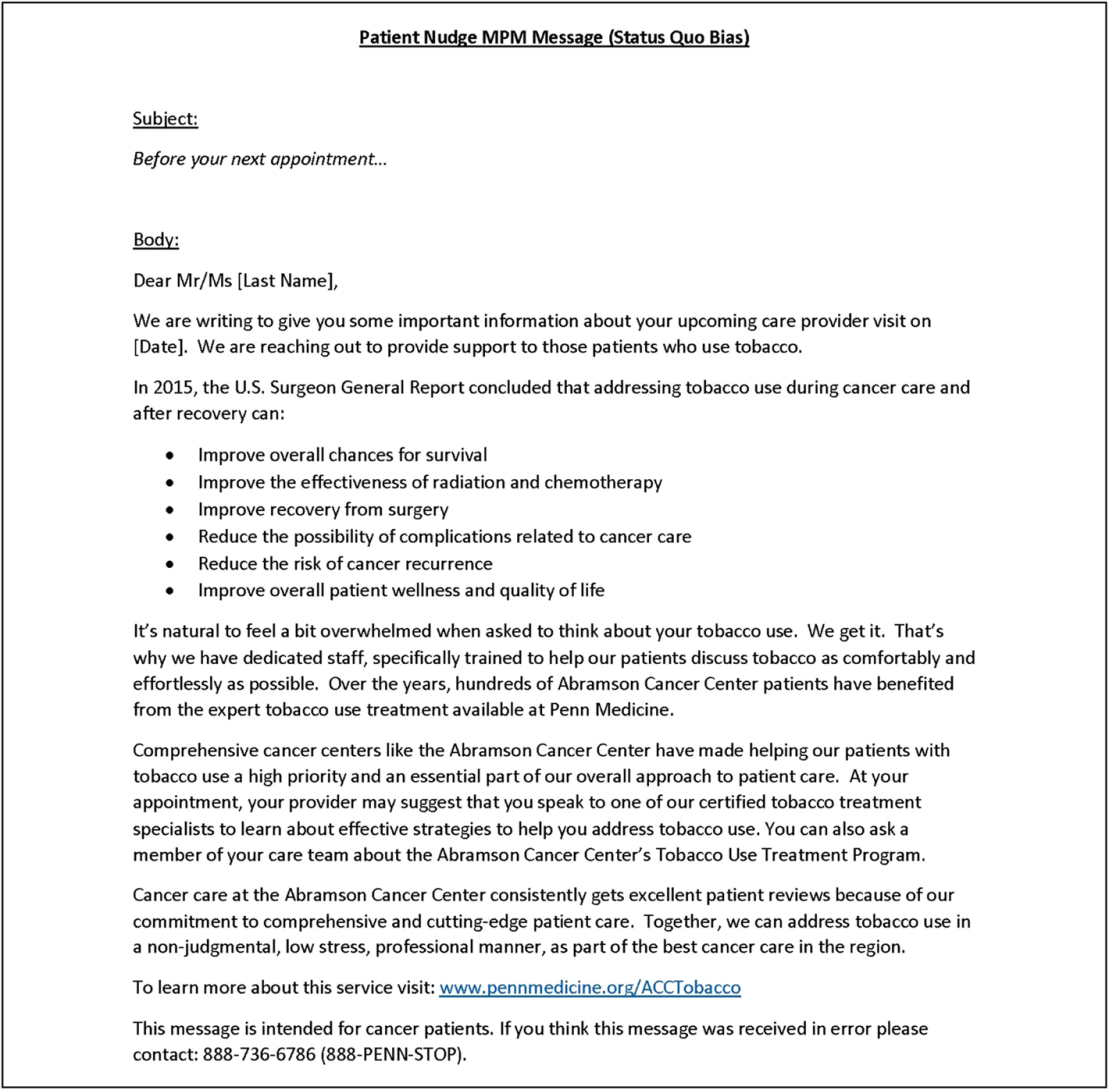
Patient nudge received via myPennMedicine prior to their subsequent visit

During the subsequent visit, clinicians randomized to the clinician-only arm receive a care guidance message at the point-of-care, promoting TUT by addressing omission bias (Fig. 4). If randomized to the patient nudge only arm, patients receive a message focused on status quo bias at least 24 h prior to the subsequent visit, encouraging them to speak with their clinician about TUT (Fig. 5). The patient nudge is delivered through *myPennMedicine*, an online communication tool used by > 75% of iLab patients through which they schedule visits, complete initial check-ins, and view test results. If randomized to usual care, the subsequent visit proceeds without additional encouragement (i.e., no nudges).

Primary outcome data—the referral to TUTS—will be ascertained from the EMR. Secondary outcome data derived from patients referred to TUTS will be collected by trained staff by telephone, via an initial counseling call within 7–10 days of the referral and a 90-day follow-up call. For aim 2, data will be ascertained through the EMR (patients) and a baseline survey (clinicians). For aim 3, data will be collected through semi-structured interviews, conducted after completion of the implementation trial to reduce contamination, and will be digitally recorded and transcribed. Such mixed-methods approaches to identify moderators and mechanisms underlying implementation strategies may help to advance causal theory and mechanisms through which implementation strategies operate in the field of implementation science [36]. Informed by the Consolidated Framework for Implementation Research (CFIR) [37], the clinician survey and patient and clinician interviews will assess inner and outer setting factors associated with responses to the nudges. The clinician survey will be conducted electronically via REDcap, with a completion goal of > 60% of the clinician sample. For our semi-structured interviews, we will interview approximately 30 patients and 30 clinicians, stratifying sampling of patients to achieve maximum variation across indices related to health equity (e.g., race/ethnicity, neighborhood-level poverty, and rurality).

### Interventions: content and delivery

Nudges have been designed to improve TUTS referral and TUT engagement through use of normative messages addressing clinician and patient biases.

#### Clinicians nudge

Findings from our preliminary work examining physician preferences toward TUT revealed a strong preference for interventions perceived to be effective [38]. These findings led us to examine the role of clinician biases regarding treatment success probabilities under conditions of uncertainty [39]. We showed that strategies minimizing well-established cognitive biases such as omission bias—the tendency to focus on the potential harm of action more than that of inaction—are more successful at changing physician behavior than strategies that solely aim to increase knowledge of TUT service availability [40].

All sites use Epic (Epic Systems Corporation, Verona, WI) to deliver care, or an interoperable system. Epic BPA deployment is modifiable, as we have already done within the iLab [18]. Upon opening the Epic Order tab at a patient’s next medical visit after the index visit, clinicians will receive the implementation strategy, placed directly over the order interface (Fig. 3). The clinician will be required to “acknowledge” or “opt-out” when presented with the order. Opting-out will require clinicians to acknowledge a reason for opt-out using a checklist or free text. Acknowledging the order places the referral to the TUTS.

#### Patient nudge

Likewise, status quo bias—tending to stick with a current choice even if better alternatives exist—can reduce patient willingness to engage in TUT [41]. The patient message, shown in Fig. 4, will include information specific to the upcoming appointment with the oncology clinician.

### Measures

#### Baseline measures

Collected through the EMR and a clinician survey, we will assess patient-level (age, sex, race/ethnicity, type of health insurance, cancer type, geocoded area as a proxy for neighborhood-level socioeconomic status, and history of prior tobacco cessation pharmacotherapy) and clinician-level (years in practice, patient panel size, and the prevalence of patient smoking in the patient panel) data. The clinician survey will assess distal constructs (e.g., organizational learning) and proximal constructs (e.g., perceived self-efficacy to discuss and help patients with tobacco cessation), given findings that these constructs are important for implementation [42].

We will also collect practice-level data: setting (community vs. hospital-based), urban vs. non-urban location, and health insurance mix. These data will be used to describe the sample of patients and clinicians participating in this study and to address aim 2.

#### Outcome measures

The primary measure of implementation is penetration (TUTS referral rate), defined as the number of TUTS orders signed, divided by the total number of submitted orders (i.e., 1-cancellation rate). Secondary outcome measures of implementation include treatment engagement defined as the number of patients who receive FDA-approved medication or behavioral counseling for tobacco use, patient quit attempts, and patient abstinence rate. Established guidelines state that real-world pragmatic, population-based trials such as this do not require biochemical verification of abstinence while including it could introduce significant bias [43]. Note, we will continue to monitor assessment rate (defined as the number of times the index visit BPA is answered divided by the total number of times it fires) to ensure the intervention does not reduce assessment of tobacco use status (baseline rate = 90%). These measures will be used primarily to assess aim 1.

#### Qualitative assessment

The semi-structured interviews will assess contextual factors across the five CFIR domains to understand patient and clinician experiences with and responses to the nudges. Among clinicians, a core interview guide comprised of semi-structured questions will be used to assess multilevel conditions and processes related to implementation of TUTS. Specific questions will probe clinicians about key barriers and facilitators of nudges for referral to TUTS. Among patients, questions will probe participants about reactions to the nudge and referral and, in line with our health equity lens, we will add questions about social and structural factors that may contribute to health inequities such as experiences of racism, discrimination, medical mistrust, perceived health care access, and language barriers. In addition to semi-structured interview questions, all patient and clinician interview participants will complete a brief questionnaire that assesses demographics and beliefs and behaviors related to tobacco cessation support (e.g., internalized stigma of smoking, barriers to providing cessation support [15, 44], referral patterns).

### Statistical analysis

#### Sample size and power (aim 1)

Based on our preliminary data, we anticipate including 900 smoking patients (based on prevalence estimate of ~ 7% among 13,000 patients screened over a 1-year study period), nested within clinicians. Analysis will use the first TUTS order generated for each patient/physician combination. Data are clustered within clinician, and the exchangeable correlation observed from other studies is small (0.07). We calculated power requirements by simulation using Stata 15, assuming a logistic regression model fitted using generalized estimating equations (GEE), and found our sample gives us 80% power to detect 11% improvement in our primary outcome (e.g., from current 34% referral rate to 45%), using a two-sided type 1 error rate of 5%, for planned comparisons between usual care and each nudge arm. The effect of the combined nudge arm is expected to be larger than each individually, indicating at least 80% power to detect probable effects for the comparison between usual care and the combined nudge arm.

#### Analysis plan

For aim 1, we will analyze all binary outcomes using logistic regression with GEE. The study design is factorial, and models will contain binary predictor terms for clinician and/or patient nudges. We will also include adjustments for time in months, fixed effects for site, and random effects for clinician cluster. We will control for type 1 error inflation by hierarchical testing, starting with the overall model significance, followed by effect of clinician nudge, followed by patient nudge. Once we have fitted the main effects model, we will test for interaction between clinician and patient nudge and retain that interaction term if significant (alpha = 5%). For aim 2, variability in these outcomes by treatment arm and moderators (particularly health equity variables) will be assessed using interaction terms within logistic regression models. We will fit an adjusted logistic regression model using the same approach described in the primary analysis. Covariates of interest available through the EMR and data collected in our clinician survey will be added to the model, including patient-level (e.g., cancer type), clinician-level (e.g., years in practice), and practice-level (e.g., community vs. hospital-based) data. We will explore implementation determinants collected within the baseline survey of clinician and organization characteristics, such as clinician biases and implementation climate, as well as moderators of implementation effectiveness.

For aim 3, we will use convergent mixed-methods analysis to help identify *for whom* implementation strategies are most effective, including among patients more likely to experience inequities, and to identify *how* strategies might work (i.e., mechanism of change) [45, 46] Informed by CFIR, we will identify contextual conditions (e.g., inner setting) and implementation conditions (characteristics of specific implementation strategy and process) shaping response to patient and clinician nudges. The constant comparative method, guided by grounded theory [47, 48], will be used to deductively code a priori domains of interest (guided by CFIR domains, including biases from behavioral economics) and to inductively explore emergent themes. We will triangulate these qualitative data with other quantitative data collected in the trial (e.g., trial outcomes, structured questionnaire data). These coded data will serve as inputs to assess multilevel mechanisms shaping nudge effectiveness across our trial and Penn ISC3.

### Project activities

The traditional research paradigm follows a phased approach where projects proceed from original idea through conceptual design to a pilot phase that may include a small, randomized trial to test feasibility. This research then graduates to a fully powered randomized trial, locked into a protocol until the last participant completes the study. This approach can be slow, but it produces highly credible answers to high-stakes questions. Leveraging our expertise in Rapid Cycle Approaches (RCA) [49, 50], we have engaged in preliminary research activities to de-risk and optimize our implementation strategies and methods so that the nudges we test, and the methods used to deliver them, in our trial, are informed by relevant stakeholder experience. We have optimized the framing of the nudges and critical aspects of the study design using RCA to ensure face validity and maximum effect. This work is summarized below and in Table 1.

**Table 1:** Rapid cycle approaches to validate nudges and optimize clinical work flow

Domain	Initial approach	Iterative work	Output
Clinician nudge	Best practice alert (BPA) identifying patient tobacco use and potential referral to tobacco use treatment. Key questions: -Specific message content -Format and timing of alert -Pre-selected referral -Best method to understand why a referral order may not be appropriate	Method: Usability testing with clinician end-users, with specific questions focused on understanding of what the system was trying to convey, key action(s) to be taken, likes/dislikes of the prototype, and any missing elements Key feedback: -Overall satisfaction with simple and concise design -Alert should be available when first opening the clinical encounter -Pre-select desired outcome	Clinician best practice alert created as an opt-in default, options for accountable justification, and guidance for when in the clinical encounter the BPA should appear.
Patient nudge	Informational message describing importance of tobacco use treatment during cancer care and available evidence-based treatment options Key questions: - Best method to deliver message (for example, via text message, patient portal, or traditional mail)	Method: Focus group comprised of cancer patients and caregivers viewed the message and provided responses to open-ended questions about its potential impact. Options for how the messages should be delivered were also reviewed and feedback on mode and timing was ascertained. Key feedback: -Send message through patient portal -Suggested wording improvements to clarify treatment options	Deliver patient nudge via patient portal with key wording changes
Identifying cancer patients who use tobacco	BPA prompting key staff to inquire about tobacco use Key questions: Which staff were best positioned to inquire about tobacco use?	Method: The BPA to assess patient tobacco use was initiated to evaluate compliance, trial randomization, and potential contamination, but with the nudges enacted in silent mode. Key feedback: We failed to include key staff conducting these assessments across several clinical sites who were not included in our original study.	Extend BPA to the full spectrum of staff responsible for initial patient contact

#### Clinician nudge

Our initial work centered on the development of the clinician nudge. This work included the refinement of the message content and the determination of the structure (format, timing) of the BPA embedded into the clinical chart. Once prototypes were devised, they were presented to a small group of clinicians for usability testing. Clinicians were asked to rate the effectiveness of the prototypes and provide open-ended responses concerning methods for refinement. The clinician nudge design to be used in this trial reflects the input and recommendations of this group, including an opt-in default, options for accountable justification, and guidance for when in the clinical encounter the BPA should appear.

#### Patient nudge

Likewise, we have conducted initial formative work to devise the patient nudge to be used in this trial. Again, this work involved efforts to optimize the format, content, and timing of the message. Once a prototype was developed, a focus group comprising cancer patients and caregivers viewed the message and provided responses to open-ended questions about its potential impact. Options for how the messages should be delivered were also reviewed and feedback on mode and timing was ascertained. This pre-testing led to our decision to use *myPennMedicine* to deliver the patient nudge and yielded the specific wording of the nudge to address the target patient bias.

#### Informatics

Once the nudge content, format, and structure were devised, we engaged in formative testing of a variety of clinical workflows and intervention structures to ensure feasibility and trial integrity. The BPA to assess patient tobacco use was initiated to evaluate compliance, trial randomization, and potential contamination, but with the nudges enacted in silent mode. This process demonstrated that compliance with the tobacco use assessment at the patient index visit was consistent with our past studies [18] but that we had failed to include key staff conducting these assessments across several clinical sites who were not included in our original study. This formative work also helped us determine that we needed to include a 2-week window to allow the clinician handling this index visit time to complete the encounter; otherwise, the clinician nudge will erroneously fire. We have retested our system to ensure that the index visit BPA and the trial randomization and nudges delivery are working correctly.

## Discussion

This study will be the first in the oncology setting to compare the effectiveness of nudges to clinicians and patients, both head-to-head and in combination, as implementation strategies to improve TUTS referral and engagement. It builds upon our prior work and targets biases among both clinicians and patients, addressing known barriers to tobacco cessation in this high-risk population. We expect the study to yield essential insights into the effectiveness of nudges as an implementation strategy to speed the uptake of high-value evidence-based TUT within cancer care, and to advance our understanding of the multilevel contextual factors that drive response to these strategies. These results will lay the foundation for how cancer care settings can ensure that patients with cancer who smoke are engaged with evidence-based TUT and may lead to a future larger clinical trial focused on scaling-up this approach across other cancer centers involved in the C3i and other cancer settings in order to maximize the impact of oncology care on patient health outcomes. We also foresee the potential of augmenting our EMR-based behavioral economic implementation strategies with other implementation strategy approaches such as improving leadership effectiveness to enhance organizational culture and climate [51].

There are key strengths and limitations of this work. The main strength of our study lies in the potential for these implementation strategies to be both highly impactful for individuals with cancer who smoke within our health system as well as highly generalizable to other clinical settings and systems. If found to be effective, our strategy would be simple to export to other EMR systems and would shed significant light on both clinician and patient factors affecting decision-making about treating tobacco use. Our design uses the strengths of a pragmatic trial while accounting for potential interaction between dual agents in the decision to engage in tobacco use treatment. Our outcomes focus on behaviors that are generally the result of a negotiated plan between clinician and patient. As such, one key limitation is that clinician decision-making may be influenced by the strategy but remain unmeasured by our protocol (e.g., patient refuses). Also, given the multidisciplinary nature of cancer care, the potential for confounding due to contamination is high (i.e., patient behaviors influenced by multiple clinicians over multiple visits). Nonetheless, we will be able to identify if the implementation strategy is ineffective and contextual qualitative data will shed light on this. Finally, it is also possible that this approach may add to clinician fatigue. However, our team has a track record of success building EMR tools, and, by grounding this work in approaches that incorporate regular end-user and stakeholder feedback plus RCA, our design process will maximize the likelihood that the approach is usable and effective [52].

## Data Availability

Not applicable.

## Abbreviations

TUT: Tobacco use treatment
NCI: National Cancer Institute
C3i: Cancer Center Cessation Initiative
EMR: Electronic medical record
TUTS: Tobacco Use Treatment Service
SES: Socioeconomic
ISC3: Implementation Science Center in Cancer Control
iLab: Implementation Lab
BPA: Best Practice Alert
CFIR: Consolidated Framework for Implementation Research
